# Knowledge and Self-Protective Practices Against COVID-19 Among Healthcare Workers in Vietnam

**DOI:** 10.3389/fpubh.2021.658107

**Published:** 2021-10-28

**Authors:** Anh Ngoc Nguyen, Xuan Thi Thanh Le, Nhung Thi Kim Ta, Danny Wong, Nguyen Thao Thi Nguyen, Huong Thi Le, Thao Thanh Nguyen, Quan Thi Pham, Quynh Thi Nguyen, Quan Van Duong, Anh Mai Luong, David Koh, Men Thi Hoang, Hai Quang Pham, Thuc Minh Thi Vu, Giang Thu Vu, Carl A. Latkin, Cyrus S. H. Ho, Roger C. M. Ho

**Affiliations:** ^1^School of Preventive Medicine and Public Health, Hanoi Medical University, Hanoi, Vietnam; ^2^School of Nursing and Health Professions, University of San Francisco, San Francisco, CA, United States; ^3^Duke School of Medicine, Duke University, Durham, NC, United States; ^4^Vietnam Health Environment Management Agency, Ministry of Health, Hanoi, Vietnam; ^5^Pengiran Anak Puteri Rashidah Sa'adatul Bolkiah (PAPRSB), Institute of Health Science, Universiti Brunei Darussalam, Gadong, Brunei; ^6^SSH School of Public Health, National University of Singapore, Singapore, Singapore; ^7^Institute for Global Health Innovations, Duy Tan University, Da Nang, Vietnam; ^8^Faculty of Medicine, Duy Tan University, Da Nang, Vietnam; ^9^Institute of Health Economics and Technology, Hanoi, Vietnam; ^10^Center of Excellence in Evidence-Based Medicine, Nguyen Tat Thanh University, Ho Chi Minh City, Vietnam; ^11^Bloomberg School of Public Health, Johns Hopkins University, Baltimore, MD, United States; ^12^Department of Psychological Medicine, Yong Loo Lin School of Medicine, National University of Singapore, Singapore, Singapore; ^13^Department of Psychological Medicine, National University Health System, Singapore, Singapore; ^14^Institute for Health Innovation and Technology (iHealthtech), National University of Singapore, Singapore, Singapore

**Keywords:** COVID-19, knowledge, practice, national lockdown, Vietnam

## Abstract

**Background:** In middle-income countries such as Vietnam, where healthcare resources are already constrained, protecting healthcare workers (HCWs) is essential for ensuring the sustainability of COVID-19 response in Vietnam. This study was conducted to assess the knowledge and practices regarding the prevention of the COVID-19 among the HCWs in Vietnam to identify the ways of disseminating information to maximize the safety of these essential workers.

**Methods:** An online cross-sectional study, using respondent-driven sampling, was conducted in Vietnam with 742 participants within 2 weeks. The validity of the questionnaire was examined by exploratory factor analysis. Descriptive statistics were used to identify the level of knowledge and practices among the HCWs to prevent the COVID-19. Inferential statistics and regression modeling were used to identify the associated factors with results.

**Results:** Vietnamese HCWs had a high level of knowledge with more than 75% of the participants demonstrating awareness of all the modes of transmission aside from air. The mean knowledge score was 3.7 ± 0.8 (range 1–5). Nearly all the participants relied on the Ministry of Health (98.3%) and the internet (95.5%) for information regarding the COVID-19. The participants endorsed a moderately high level of self-protective practices with mean scores of 4.2 and 3.6 (band score 1–5) for the precautionary and psychological measures, respectively. Nurses were more likely to practice the precautionary measures than doctors and the HCWs at the central level were more likely to practice the psychological measures than those at the district level.

**Conclusion:** Future education initiatives should consolidate the latest literature in an accessible format, focusing initially on the gaps of knowledge regarding aerosol transmission. These initiatives should primarily focus on the doctors, especially those in emergency and intensive care departments.

## Introduction

In December 2019, China first reported the cases of pneumonia caused by SARS-CoV-2, the beginning of the global coronavirus disease 2019 (COVID-19) pandemic (1, 2). Since the initial outbreak, the healthcare workers (HCWs) have assumed an essential role in defending the health of the population and are at heightened risk of contracting infection (3). Italy, an early epicenter of the global COVID-19 pandemic, lost 151 doctors and more than 40 nurses to the COVID-19 by the end of April 2020 (4). In Northern Italy, the infection rate of the HCWs was documented to be about 20% (5). The US, a later epicenter, was found to have 62,344 confirmed COVID-19 cases and 291 deaths among the HCWs by late May 2020 (6).

In contrast to Italy and the US, the effects of the COVID-19 pandemic in Vietnam have been minimized with proactive interventions including early detection, timely isolation, and strict adherence to social distancing (5, 7–9). As a part of the prevention strategy in Vietnam, the Vietnamese government also implemented a partial 15-day national lockdown, closing restaurants and public spaces, stopping public transportation in high-risk areas, and restricting travel (10). As a result, on September 23, 2020, there have been only a total of 1,069 reported COVID-19 cases and 35 deaths in the country, a success story in terms of pandemic containment (11). In July 2020, only four Vietnamese HCWs have become infected (12). However, as of 12:56 GMT, August 28, 2021 (more than 1 year after the time of the study), a total of 422,469 reported COVID-19 cases and 10,405 deaths in Vietnam (13). Therefore, efforts to prevent HCW infection are especially crucial in Vietnam given preexisting constraints on the healthcare resources.

In addition to public health interventions, personal hygiene practices such as handwashing and adherence to personal protective equipment (PPE) are essential in efforts to prevent transmission. Previous studies have described the shortages of PPE globally (14–16). Vietnam is no different and many health workers have had to use non-medical grade face masks. In view of this, new recommendations have been released to guide mask allocation and usage (10, 17). However, HCW safety requires not only access to PPE but also sufficient knowledge and practices to prevent the COVID-19 transmission. Currently, studies on the latter among the HCWs in Vietnam are lacking.

Previous research on the knowledge and practices of HCWs in China found that 89% of the HCWs had sufficient knowledge and 89.7% followed correct practices for disease prevention (18). A similar study in Italy found overall adequate knowledge of the COVID-19 control measures among the HCWs (19). In Vietnam, one study found that the HCWs had a good understanding of the COVID-19 with a mean knowledge score of 8.17 (range 4–10). These data were collected from a single hospital in Ho Chi Minh City, Vietnam (20). Utilizing an online questionnaire to reach more participants, this study aims to characterize knowledge and practices of COVID-19 prevention among the HCWs across Vietnam.

By examining the sources of information of the HCWs, this study aims to identify ways to disseminate information to the HCWs. Moreover, due to the heterogeneity of the HCW population, this study also seeks to identify characteristics associated with gaps in knowledge and practices of COVID-19 prevention to guide the opportunities for further education.

## Methods

### Study Setting and Respondents

A cross-sectional study was conducted on 2 weeks of early April 2020 during the partial national lockdown in Vietnam. At this point during the epidemic in Vietnam, more than 60% of the COVID-19 cases were brought from foreign countries including Hubei, Wuhan, and China, the initial epicenter of the COVID-19 pandemic. The eligibility criteria for the participants were the following: (1) occupation as an HCW, (2) agreement to participate through an online informed consent, and (3) ability to access the web-based questionnaire.

### Sample and Sampling

Respondent-driven sampling was used to recruit the respondents. At the beginning of the recruitment process, a core group at the Hanoi Medical University was created. Members of this group were chosen based on the high likelihood of knowing HCWs at various hospitals throughout Vietnam. They were also selected to reflect a diverse range of characteristics including gender, age, and occupation. The core group sent the questionnaire link to their close contacts *via* platforms such as Facebook or Zalo. Respondents were able to access the questionnaire on computers, tablets, and smartphones, and they were also asked to invite the other Vietnamese HCWs to participate. A total of 742 HCWs participated in this study, which consisted of staff at the hospitals and the medical universities throughout all the 63 provinces.

### Instruments and Measures

An online questionnaire was created by using the SurveyMonkey platform, which collected the data on the demographics and occupation of the participants along with knowledge and practices of the COVID-19 prevention. The questionnaire in Vietnamese was developed by the public health experts of the Institute of Preventive Medicine and Public Health, Hanoi Medical University. It was based on the questionnaires regarding the perception of risk of the HCWs and the preventive measures for SARS in Singapore. Demographic characteristics consisted of information such as age, gender, marital status, religion, ethnic group, education level, and home environment. Occupational characteristics included job title and current work status.

Finally, to evaluate for the knowledge and practices of the COVID-19 prevention, the questionnaire asked participants to rate the statements on knowledge and self-protective actions to prevent the COVID-19 on a 5-point Likert scale in which 1 representing “strongly disagree” and 5 representing “strongly agree.”

### Data Analysis

The collected data were analyzed by using STATA 15.0 (StataCorp LP, College Station, Texas, United States). Characteristic data including mean, SD, frequency, and percentage were examined by using descriptive statistics. Inferential statistics were used to compare the HCWs at the central hospitals, provincial hospitals, and other hospitals. The Fisher's exact or chi-squared tests were used for the qualitative variables and ANOVA or Kruskal–Wallis test were used for the quantitative variables. A multivariable linear regression model was used to identify the factors associated with knowledge and practices of COVID-19 prevention. The stepwise forward selection was utilized to obtain reduced models with a log-likelihood ratio test at a *p*-value of 0.2. Statistical significance was set at a *p* < 0.05.

### Ethical Consideration

This project was ethically approved by the Review Committee at the Institute of Preventive Medicine and Public Health, Hanoi Medical University on March 28, 2020. The research purpose and informed consent were provided on the web-based survey before participation. Participation was anonymous and voluntary and respondents could withdraw from this study at any point.

## Results

Table 1 shows the demographic characteristics of the participants in this study. Most of the respondents (62.9%) were females and they worked in Northern Vietnam (71.8%). More than half of the respondents were doctors (55.4%) and they were educated at the lower university level (59.4%). Regarding department, 16.8% of the participants worked in radiology, scientific laboratories, or clinic; 14.0% of the participants worked in the administrative offices; and only 4.7% of the participants worked in infectious disease and infection control.

**Table 1:** Participant demographics.

**Characteristics**	**Geographical hospital divisions**								***p*** **-value**
	**Central level**		**Provincial level**		**Others**		**Total**		
	***n***	**%**	***n***	**%**	***n***	**%**	***n***	**%**	
**Total**	167	31.6	159	30.1	203	38.4	529	100.0	
**Region**									
Northern	151	90.4	85	53.5	144	70.9	380	71.8	< 0.01
Central	11	6.6	63	39.6	38	18.7	112	21.2	
South	5	3.0	11	6.9	21	30.3	37	7.0	
**Gender**									
Male	63	37.7	56	35.2	72	35.5	191	36.1	0.87
Female	104	62.3	103	64.8	131	64.2	338	62.9	
**Marital status**									
Single/Separated/Widowed	32	19.2	37	23.3	42	20.7	111	21.0	0.66
Married	135	80.8	122	76.7	161	79.3	418	79.0	
**Living with**									
Family/friends	154	92.2	147	92.5	189	93.1	490	92.6	0.94
Alone	13	7.8	12	7.6	14	6.9	39	7.4	
**Education**									
University and lower	71	42.5	102	64.2	141	69.5	314	59.4	< 0.01
>University	96	57.5	57	35.9	62	30.5	215	40.6	
**Occupation**									
Doctor	104	62.3	76	47.8	113	55.7	293	55.4	< 0.01
Nurse	30	18.0	54	34.0	40	19.7	124	23.4	
Others	33	19.8	29	18.2	50	24.6	112	21.2	
**Department**									
Emergency-intensive care	6	3.6	20	12.6	17	8.4	43	8.1	< 0.01
Internal medicine	28	16.8	20	12.6	20	9.9	68	12.9	
Surgery-obstetrics-pediatrics	24	14.4	22	13.8	20	9.9	66	12.5	
Radiology-scientific laboratory-clinic	34	20.4	16	10.1	39	19.2	89	16.8	
Administrative offices	14	8.4	32	20.1	28	13.8	74	14.0	
Infectious disease-infection control	10	6.0	6	3.8	9	4.4	25	4.7	
Preventive medicine-public health-nutrition	29	17.4	5	3.1	33	16.3	67	12.7	
Others	22	13.2	38	23.9	37	18.2	97	18.3	
	**Mean**	**SD**	**Mean**	**SD**	**Mean**	**SD**	**Mean**	**SD**	***p*** **-value**
Age	35.8	9.1	34.7	8.3	36.4	9.0	35.7	8.9	0.17
Durationof career (years)	10.5	8.5	10.6	8.2	11.5	8.9	10.9	8.6	0.54

Table 2 summarizes the knowledge, source of information, and practices regarding COVID-19 prevention among the participants. The majority of the participants knew that close contact with colleagues who have cared for the patients with the COVID-19 (79.5%), directly caring for the patients with the COVID-19 (75.6%), or touching surface containing nasal or salivary secretions of the patients with the COVID-19 (75.6%) could increase the risk of developing the COVID-19. Less than half of the participants (41.4%) knew that the SARS-CoV-2 virus could be transmitted by air. On a scale of 1–5, the average knowledge score of the respondents was 3.7 (SD = 0.8). The internet (95.5%) and television (83.6%) were the most popular media channel for seeking information. Moreover, the Ministry of Health was the most widely used source of information with 98.3% of all the participants obtaining information. Regarding the practices, the average score was relatively high ranging from 3.3 to 4.5. Practices with the highest rates of engagement were seeking information about the COVID-19, complying with the Ministry of Health preventive recommendation, and avoiding crowded places (average scores 4.4, 4.5, and 4.4, respectively).

**Table 2:** Knowledge, sources of information, preferences for training platform, and practice on the transmission and self-prevention against the coronavirus disease 2019 (COVID-19).

**Characteristics**	**Level of hospital**								***p*** **-value**
	**Central level**		**Provincial level**		**Others**		**Total**		
	***n***	**%**	***n***	**%**	***n***	**%**	***n***	**%**	
**Knowledge of transmission**									
Air	64	52.5	65	60.8	90	57.7	219	26.9	0.44
Close contact with COVID-19 patients	88	72.1	77	72.0	1,126	80.8	291	75.6	0.15
Colleagues with contact with COVID-19 patients	91	74.6	84	78.5	131	84.0	306	79.5	0.15
Surface with nasal/throat secretions of COVID-19 patients	93	76.2	83	77.6	115	73.7	291	75.6	0.76
**Source of information about COVID-19**									
WHO	118	70.7	93	58.5	115	56.7	326	61.6	0.01
Ministry of Health	164	98.2	157	98.7	199	98.0	520	98.3	0.92
CDC	103	61.7	82	51.6	115	56.7	300	56.7	0.18
University/ Hospital	119	71.3	90	56.9	101	49.8	310	58.6	< 0.01
Colleague	109	65.3	99	62.3	121	59.6	329	62.2	0.54
Friends	70	41.9	65	40.9	77	37.9	212	40.1	0.72
Others	19	11.4	14	8.8	28	13.8	61	11.5	0.34
**Mass media channel for information seeking**									
Internet	162	97.0	151	95.0	192	94.6	505	95.5	0.50
Television	130	77.8	135	84.9	177	87.2	442	83.6	0.05
Radio	55	32.9	61	38.4	90	44.3	206	38.9	0.08
Newspaper	95	56.9	96	60.4	124	61.1	315	59.6	0.69
Other	14	8.4	9	5.7	14	6.9	37	7.0	0.63
**Information about COVID-19 that you want to know more**									
Nothing	31	18.6	19	12.0	37	18.2	87	16.5	0.19
Epidemiology	73	43.7	65	40.9	86	42.4	224	42.3	0.88
Infection control and prevention	90	53.9	89	56.0	102	50.3	281	53.1	0.54
Disease management	80	47.9	74	46.5	83	40.9	237	44.8	0.35
PPE use	61	36.5	71	44.7	81	39.9	213	40.3	0.32
**Forms of training desired**									
Direct training	27	16.2	39	24.5	39	19.2	105	19.9	0.16
Online lecture	83	49.7	61	38.4	81	39.9	225	42.5	0.07
Online seminar/webinars	49	29.3	38	23.9	47	23.2	134	25.3	0.35
Phone application	66	39.5	67	42.1	63	31.0	196	37.1	0.07
Text documents	53	31.7	65	40.9	54	26.6	172	32.5	0.02
	**Mean**	**SD**	**Mean**	**SD**	**Mean**	**SD**	**Mean**	**SD**	***p*** **-value**
**Adequate knowledge of transmission** (band score 1–5)	3.7	0.8	3.7	0.7	3.7	0.9	3.7	0.8	0.63
**Self-protective actions**									
Seek information about COVID-19	4.5	0.6	4.4	0.7	4.4	0.9	4.4	0.7	0.48
Comply with recommendations and preventive measures of the Ministry of Health	4.6	0.6	4.5	0.6	4.4	0.8	4.5	0.7	0.46
Avoid crowded places	4.5	0.6	4.5	0.6	4.3	0.9	4.4	0.7	0.17
Avoid contact with colleagues at risk of COVID-19 exposure	3.7	0.9	3.6	0.9	3.4	1.0	3.6	1.0	0.02
Nutritional supplements, vitamins	4.0	0.8	4.0	0.8	4.0	0.9	4.0	0.8	0.58
Exercise regularly	4.0	0.8	4.0	0.8	3.9	0.9	4.0	0.8	0.80
Try not to think about the risks of COVID-19	3.3	1.0	3.2	1.0	3.3	1.0	3.3	1.0	0.41
Stay positive and convince yourself that you will not be infected with COVID-19	3.9	0.8	3.8	0.9	3.8	0.8	3.8	0.8	0.73
Accept the risk of COVID-19 infection	3.8	0.7	3.7	0.8	3.7	0.8	3.7	0.7	0.30

The construct validity of self-protection from the COVID-19 is displayed in Table 3. Self-protective actions were subdivided into two categories: precautionary measures to prevent disease transmission and psychological measures to promote mental well-being. Table 4 demonstrates the values of Cronbach's alpha for those categories that were 0.83 and 0.55, respectively.

**Table 3:** Measures taken to self-protect from COVID-19.

**I have prevented COVID-19 when I**	**Strongly agree**		**Precautionary measures**	**Measures toimprovepsychological well-being**
	***n***	**%**		
Comply with Ministry of Health recommendations and preventive measures	298	56.3	0.92	
Avoid crowded places	273	51.6	0.90	
Seek information about COVID-19	267	50.5	0.88	
Nutritional supplements, vitamins	155	29.3	0.65	
Exercise regularly	143	27.0	0.60	
Avoid contact with colleagues at risk of exposure to COVID-19	83	15.7	0.42	
Stay positive and convince yourself that you will not be infected with COVID-19	95	18.0		0.78
Accept the risk of COVID-19 infection	50	9.5		0.54
Try not to think about the risks of COVID-19	46	8.7		0.77
**Cronbach's alpha**			0.83	0.55
**Mean**			4.2	3.6
**SD**			0.6	0.6

**Table 4:** Associated factors of the knowledge and practices against the COVID-19.

**Factors**			**Self-protection from COVID-19**			
	**Adequate knowledge ofSARS-CoV-2transmission**		**Precautionary measures**		**Measures to improve psychological well-being**	
	**Coef**.	**95% CI**	**Coef**.	**95% CI**	**Coef**.	**95% CI**
**Region (vs. Northern)**						
Central					0.12	−0.04; 0.28
South	1.47^**^	1.08; 2.00				
**Gender (vs. Male)**						
Female			−0.12^*^	−0.24; 0.00		
**Education (vs. University and lower)**						
> University			0.18^***^	0.05; 0.30		
**Occupation (vs. Doctor)**						
Nurse			0.18^**^	0.02; 0.34	0.25^***^	0.10; 0.40
Others	0.81^*^	0.66; 1.00				
**Level of hospital (vs. Central level)**						
District health center					−0.18^**^	−0.31; −0.04
Others			−0.12	−0.29; 0.05		
**Department (vs. Emergency-Intensive care)**						
Surgery-obstetrics-pediatrics	0.74^**^	0.57; 0.97	−0.14	−0.33; 0.04		
Administrative offices	0.75^**^	0.58; 0.96				
Infectious disease-infection control	0.76^**^	0.58; 0.98				
Preventive medicine-public health- nutrition	0.78	0.54; 1.12	−0.26^*^	−0.52; 0.00		
Others	0.73^***^	0.58; 0.92				
**Adequate knowledge of transmission**			0.20^***^	0.13; 0.27	0.07^*^	−0.00; 0.15
**Source of information about COVID-19**						
CDC					0.09	−0.03; 0.22
University/Hospital			0.1	−0.02; 0.22		
**Mass media channel for information seeking (vs. television)**						
Internet	1.19	0.92; 1.55			0.57^***^	0.22; 0.92
Radio			0.13^**^	0.01; 0.25		
Newspaper	0.84^**^	0.70; 1.00			−0.13^**^	−0.26; −0.00
Other	1.36^*^	0.99; 1.86				
**Aspect of COVID-19 that respondents wanted to know more about**						
Epidemiology COVID-19			0.11^*^	−0.01; 0.22	0.35^***^	0.22; 0.48
Case management	1.18^*^	0.99; 1.41				
Using PPE					−0.21^***^	−0.34;−0.08

*** *p < 0.01;*

** *p < 0.05;*

* *p < 0.1.

Table 4 represents the relationships between the demographic characteristics and source of information with the perceived knowledge and practices of the respondents against the COVID-19. Aside from those who worked in the preventive medicine, public health, and nutrition departments, we found that the HCWs in all the other departments were more likely to have adequate knowledge of SARS-CoV-2 virus transmission and the COVID-19 prevention than those in the emergency and intensive care unit.

Nurses were more likely to practice precautionary measures against the COVID-19 compared to doctors (Coef. = 0.18, 95% CI = 0.02–0.34). HCWs with an education level higher than university (Coef. = 0.18, 95% CI = 0.05–0.30) and who had adequate knowledge of transmission (Coef. = 0.20, 95% CI = 0.13–0.27) were also more likely to engage in the precautionary measures than their counterparts.

In terms of psychological well-being, the HCWs at the district level health centers were less likely to engage in self-protective practices than working at the central hospitals (Coef. = 0.18, 95% CI = 0.31–0.04). Meanwhile, using the internet for information about the COVID-19 was correlated with greater implementation of the measures to improve psychological well-being (Coef. = 0.57, 95% CI = 0.22–0.92).

## Discussion

To the best of our knowledge, this study is among the first to examine both the knowledge and self-protective practices to prevent the COVID-19 among the HCWs in Vietnam. Overall, the results showed that Vietnamese HCWs have a high level of knowledge with more than 75% of the participants demonstrating awareness of all the modes of transmission aside from air. The mean knowledge score was 3.7. The HCWs in the emergency and intensive care departments had lower knowledge scores than those in nearly all other departments. Further, participants endorsed a high level of self-protective practices with mean scores of 4.2 and 3.6 for the precautionary and psychological measures, respectively. Nurses were more likely to practice the precautionary measures than doctors and the HCWs at the central level were more likely to practice the psychological measures than those at the district level.

The knowledge levels in this study are lower than those found among the HCWs in Pakistan (21) and higher than nurses in Iran (22). In comparison to a prior study conducted at a hospital in Ho Chi Minh City, Vietnam, the knowledge score in this study was slightly lower. However, with respect to transmission, a greater proportion of the participants in this study was aware of disease spread through close contact (20). Overall, the level of knowledge regarding transmission by contact with infected people and surfaces echoes findings from prior studies (23, 24). A knowledge gap on aerosol transmission was also found. It is likely due to the limited data on the aerosol transmission during the pandemic when this study was conducted. Since then, research has emerged to suggest the possibility of short-range aerosol transmission (25, 26). As such, future initiatives among the HCWs in Vietnam should focus on education regarding the COVID-19 transmission by air and its implications on self-protective clinical practices.

Compared to hospital staff in the other departments, those in emergency and intensive care units had lower knowledge about the COVID-19 transmission. Precluding them from seeking up-to-date literature about the COVID-19 may be due to the longer shifts and overall higher stress from caring for the severe patients (27–31). Given the time constraints of the HCWs, interventions should focus on consolidating information efficiently to ensure that the healthcare providers have access to updated data in the midst of high clinical demands, specifically those in emergency and intensive care units. The most popular sources of information reported by the participants were the Ministry of Health and the Internet that are reflective of findings from the previous studies (23, 32). Therefore, those mediums should be the primary focus channels of future educational campaigns. Moreover, our data emphasize the importance of keeping the Ministry of Health website routinely updated to ensure that healthcare providers have access to the latest data to guide patient care.

Regarding self-protective practices, our study found that the participants had higher levels of engagement with the precautionary measures compared with the psychological measures. This finding may represent the prioritization of physical health over mental well-being during the initial phases of the COVID-19 outbreak. The higher levels of the precautionary practices are likely attributable to the strict control measures by the Vietnamese government with significant fines for violations (33). However, as the course of the pandemic progresses and its psychological effect becomes more apparent (34), our data suggests the need for the healthcare systems to facilitate the targeted efforts to promote the mental and emotional well-being of the HCWs.

As well-documented in literature with the COVID-19 (35) and the other disease outbreaks (36, 37), knowledge scores were positively associated with practice scores. This finding emphasizes the pivotal role of education in promoting compliance with public health interventions. Nurses practiced precautionary measures at greater rates than doctors. It is likely due to the greater levels of interaction with the COVID-19 patients on a daily basis. Future interventions should continue to focus on the doctors to ensure that they are leading as team leaders. With respect to psychological measures, the HCWs at the central level hospitals had higher practice scores than those at the district level. This likely relates to greater levels of staff support available at the central level. Future interventions should ensure equitable distribution of the resources for health workers at all levels of the health system.

Our research had several limitations. A cross-sectional study had conducted at the beginning of the COVID-19 pandemic in Vietnam. Further research will be needed to identify trends in the knowledge and practices of HCWs in later phases of the COVID-19 pandemic. Second, despite a high number and diversity of the participants, our respondents were not randomly selected and are not representative of the Vietnamese HCWs population. Lastly, though the questionnaire is the first national survey to provide a glimpse into the knowledge and practices of the HCWs in Vietnam, the number of items assessed for the knowledge and practices was limited. Further, the internal validity of the psychological measures was low. Future studies will be needed to better characterize these variables, particularly the psychological practices among the HCWs to guide future interventions.

## Conclusion

This study found that the Vietnamese HCWs had a high level of knowledge and practices to prevent the COVID-19, though there was some room for improvement. Future education initiatives should focus initially on the COVID-19 virus aerosols that fill an important gap of knowledge. The Ministry of Health website should be considered for rapid dissemination of information. Doctors, especially those in emergency and the intensive care departments, should be a primary focus of these initiatives. At the same time, greater efforts will be needed to promote HCW engagement with the psychological self-protective practices, particularly among those working at the district levels.

## Data Availability Statement

The raw data supporting the conclusions of this article will be made available by the authors, without undue reservation.

## Ethics Statement

The studies involving human participants were reviewed and approved by this project was ethically approved by the Review Committee at Institute for Preventive Medicine and Public Health, Hanoi Medical University on March 28, 2020. The patients/participants provided their written informed consent to participate in this study.

## Author Contributions

AN, XL, NT, DW, HL, TN, QP, QN, QD, AL, DK, MH, HP, TV, GV, CL, CH, and RH contributed to conceptualization, writing, reviewing, and editing. NT, TN, QP, QN, QD, and MH contributed to data curation. XL, AN, NT, and HP contributed to formal analysis. XL, AN, NT, HL, AL, DK, and RH contributed to the investigation. XL, AN, NT, HP, and MH contributed to methodology. XL, AN, MH, HP, and GV contributed to project administration. XL, HL, AL, DK, TV, CL, and RH contributed to supervision. XL, NT, AN, DW, GV, and CL contributed in writing original draft. All authors contributed to the article and approved the submitted version.

## Funding

This study was funded by the Vingroup Joint Stock Company (Vingroup JSC) and supported by the Vingroup Innovation Foundation (VINIF) under the project code VINIF. 2020.COVID-19.DA03.

## Conflict of Interest

The authors declare that the research was conducted in the absence of any commercial or financial relationships that could be construed as a potential conflict of interest.

## Publisher's Note

All claims expressed in this article are solely those of the authors and do not necessarily represent those of their affiliated organizations, or those of the publisher, the editors and the reviewers. Any product that may be evaluated in this article, or claim that may be made by its manufacturer, is not guaranteed or endorsed by the publisher.
